# Fecal microbiome differs between patients with systemic sclerosis with and without small intestinal bacterial overgrowth

**DOI:** 10.1177/23971983211032808

**Published:** 2021-07-24

**Authors:** Daniel Levin, Giada De Palma, Hannah Zou, Ava Hadi Zadeh Bazzaz, Elena Verdu, Barbara Baker, Maria Ines Pinto-Sanchez, Nader Khalidi, Maggie J Larché, Karen A. Beattie, Premysl Bercik

**Affiliations:** Faculty of Health Sciences, McMaster University, Hamilton, ON, Canada

**Keywords:** Dysbiosis, microbiome, depression, small intestinal bacterial overgrowth, gastrointestinal symptoms

## Abstract

**Introduction:**

Gastrointestinal manifestations of systemic sclerosis affect up to 90% of patients, with symptoms including diarrhea and constipation. Small intestinal bacterial overgrowth is a condition associated with increased numbers of pathogenic bacteria in the small bowel. While currently unknown, it has been suggested that dysregulation of the fecal microbiota may play a role in the development of systemic sclerosis and small intestinal bacterial overgrowth.

**Objectives:**

Our study aimed to describe the fecal microbiota of patients with systemic sclerosis and compare it between those with and without a diagnosis of small intestinal bacterial overgrowth. We also compared the fecal microbiota of systemic sclerosis patients with that of healthy controls to understand the association between particular bacterial taxa and clinical gastrointestinal manifestations of systemic sclerosis.

**Methods:**

A total of 29 patients with systemic sclerosis underwent breath testing to assess for small intestinal bacterial overgrowth, provided stool samples to determine taxonomic assignments, and completed the University of California Los Angeles Scleroderma Clinical Trial Consortium Gastrointestinal Tract 2.0, which details symptoms and quality-of-life factors. Stool samples were compared between systemic sclerosis patients with and without small intestinal bacterial overgrowth, and between patients with systemic sclerosis and a healthy control cohort (n = 20), aged 18–80 years.

**Results:**

Fecal microbiome analyses demonstrated differences between systemic sclerosis patients with and without small intestinal bacterial overgrowth and differences in the diversity of species between healthy controls and patients with systemic sclerosis. Trends were also observed in anticentromere antibody systemic sclerosis patients, including higher *Alistipies indistincus* spp. levels associated with increased methane levels of breath gas testing and higher *Slakia* spp. levels associated with increased rates of fecal soiling.

**Conclusions:**

Our results suggest that changes to the fecal microbiome occur in patients with small intestinal bacterial overgrowth and systemic sclerosis when compared to healthy controls. As a cross-sectional study, the potential pathophysiologic role of an altered microbiome in the development of systemic sclerosis was not considered and hence needs to be further investigated.

## Introduction

The gastrointestinal (GI) tract is the second most common system involved in systemic sclerosis (SSc),^1^ impacting as many as 90% with the disease.^2^ Symptoms include dyspepsia, dysphagia, abdominal distension, diarrhea, constipation, and fecal incontinence, some of which may be related to small intestinal bacterial overgrowth (SIBO) in these patients. These symptoms have been shown to correlate with depressive symptoms,^3^ lower quality of life,^4^ and higher associated healthcare cost.^5^ As GI involvement is the most common cause of morbidity and the third most common cause of mortality in SSc, elucidating the poorly understood pathophysiology may help guide future therapies. Recently, an interest has developed for the role of the fecal microbiome as a contributing factor to the development of SSc.

The intestinal microbiota consists of many bacteria, all playing a role in developing both the innate and adaptive immune response. A growing body of clinical and experimental data suggest that chronic inflammatory responses induced by altered fecal microbiota contribute to the development of rheumatological disease. For instance, the development of rheumatoid arthritis (RA) has been linked to increases in *Eubacterium aerofaciens*,^6^ *Clostridium perfringens*,^7^ and *Prevotella copri.*^8^ Altered fecal microbiota has also been linked to other diseases including systemic lupus erythematosus,^9^ Sjogren’s syndrome,^10^ and SSc.^11,12^ While variability in bacterial species was observed in fecal samples of SSc patients from California (University of California Los Angeles (UCLA)) and Norway (Oslo), similarly low levels of protective *Bacteroides* spp. were seen in both cohorts.^13^ In addition, other protective organisms including *Faecalibacterium* spp. (UCLA) and *Clostridium* spp. (Oslo) were reduced, while pathogenic *Fusobacterium* spp. (UCLA) were increased. Particularly interesting was the finding that specific genera were associated with the severity of GI symptoms, which further suggests altered fecal microbiota may contribute to clinical symptoms.

SIBO is defined as an increase in the number of bacteria to over 105 colony-forming units/mL or atypical bacteria in the proximal small intestine.^14^ SIBO is common (39%) in patients with SSc, with symptoms including abdominal bloating, early satiety, diarrhea, and, when more severe, malnutrition and death.^14^ While the etiology for the development of SIBO in SSc is unknown, studies point to GI dysmotility as a potential cause.^15^ It is unclear what effect SIBO has on the colonic microbiome of patients with SSc, and how it correlates with their symptoms.

Several studies explored the potential role of SIBO in explaining differences in the fecal microbiome composition between clinical populations and healthy controls (HCs). One study found feces transplanted from SIBO+ donors resulted in bloating, diarrhea, and constipation in receiving patients.^12^ Another studied patients with and without SIBO and found that while duodenal and rectal biopsies of these patients differed, their fecal microbiomes did not.^15^ These findings suggest that SIBO, normally a proximal, dysmotility-driven issue, may have downstream effects on the microbiome in the distal bowel. In addition, anticentromere antibodies (ACA), often tested in the diagnostic workup of SSc and SIBO, have been found to have no correlation to GI involvement^16,17^ or SIBO,^14^ making the role of this antibody in predicting disease course challenging. To date, there have been no studies investigating microbiome differences between ACA-positive and ACA-negative SSc, SIBO-positive patients, and the potential role these antibodies play in the pathophysiology of SSc.

Given the high proportion of SSc patients with SIBO, exploring how proximal overgrowth affects distal symptoms (diarrhea, fecal soiling, and incontinence) in the context of the fecal microbiome is valuable and has not yet been explored. Our study aimed to (1) describe the microbiota of SSc patients in a Canadian cohort and compare these to HCs, (2) determine whether the microbiome of patients having SSc with or without SIBO are significantly different, and (3) determine whether certain bacterial taxa play a significant role in the clinical expression of GI symptoms of SSc.

## Methods

### Scleroderma patients

Patients ⩾ 18 years of age diagnosed with SSc were recruited from two rheumatology practices as part of a single-center study. Consecutive patients with and without GI symptoms were informed about the study at their clinic visit. Patients who did not have a clinic visit during our recruitment period were mailed an information package and received a follow-up phone call within 2 weeks. Those agreeing to participate were given stool sample kits. Participants were scheduled for breath testing and time to submit their fecal sample. Patients were asked to withhold medications and probiotic supplements (e.g. proton pump inhibitors, H2 blockers, laxatives, antibiotics, and antifungals (Supplementary Materials 1)) for 2 weeks prior to sample collection. The study was approved by the Hamilton Integrated Research Ethics Board (project 3788).

### Healthy controls

Fecal samples from body mass index (BMI)- and sex-matched HCs were used from a previous study (clinicaltrials.gov NCT03492333). Controls with a history of any organic disease, immune deficiency, and major abdominal surgery and those using immunosuppressants, glucocorticoids, or opioids were excluded.

### Breath samples and SIBO diagnosis

Prior to the breath test, patients received written instructions and followed a strict diet (Supplementary Materials 1) to mitigate diet as a contributing variable to breath test results. Prior to undergoing the breath test, participants were screened regarding compliance to the written instructions. Patients exhaled into a 400-mL disposable polyethylene bag, while breath samples were extracted using a 30-mL syringe. A baseline sample was taken, after which patients had 5 min to drink Trutol^®^ 75 g glucose solution. Seven more breath samples were obtained every 20 min. Breath samples were transferred from syringes into 250 mL gas-impermeable sample-holding bags that maintained the integrity of the breath samples. All breath-test kits were packed in individual sealed bags, transferred to the GI laboratory, and analyzed for hydrogen (H_2_) and methane (CH_4_) levels using the BreathTracker Digital SC model. To determine the presence of SIBO, an increase of at least 20 ppm from the baseline H_2_ reading by 90 min or a level of ⩾10 ppm for CH_4_ was required. Results were reviewed by a physician, as this is the only fully validated test in SSc.^18^

### Fecal sample collection and fecal microbiota analysis

Each patient was given a kit for stool collection, which included one plastic container, two new plastic bags, and one AnaeroGen pack (Oxoid, Nepean, ON, Canada). Patients were instructed to collect the fresh fecal sample in the plastic container, immediately place this in the plastic bag containing the AnaeroGen pack, and finally place both into another plastic bag that was kept in their fridge for up to 48 h prior to delivering it to the Rheumatology Clinic. These samples were then transported to hospital with cooler packs and kept at −80°C until analysis. Total genomic DNA was extracted as previously described.^19^ Following this protocol, amplification of the V3 region of the 16S *rRNA* gene and Illumina sequencing were performed as previously described,^19,20^. Briefly, the data were analyzed following the pipelines of dada2^21^ and QIIME2.^22^ Taxonomic assignments were performed using the RDP classifier^23^ with the Greengenes^24^ (2013) training set. Analyses were done using IIME2,^22^ MAaslin,^25^ LefSe,^26^ PICRUSt,^27^ Phyloseq package (1.28)(4) for R (3.6.1), and SPSS software v. 23. All results were corrected for multiple comparisons, allowing 5% of false discovery rate (FDR). All scripts used for the analyses are available upon request.

### GI tract symptoms

GI tract manifestations were assessed using the UCLA Scleroderma Clinical Trial Consortium Gastrointestinal Tract 2.0 (UCLA SCTC GIT 2.0). It assesses seven categories related to GI symptoms and can discriminate between self-rated severity of GI tract involvement. The higher the total GI tract score, evaluating health-related quality of life (HRQOL) and GI tract symptom severity indicates worse symptoms.

### Analyses

Analyses were performed using SPSS 23.0 software for Windows (SPSS Inc, Chicago, IL, United States), R (version 3.6.1), and GraphPad Prism. The data are presented as median (interquartile deviation (IQD)) or mean ± SEM. Statistical comparisons were performed using t-test, Mann–Whitney, or Kruskal–Wallis tests, as appropriate. Spearman’s correlations were run to assess associations between patients’ characteristics and microbiota data. To correct for multiple hypothesis testing, Benjamini and Hochberg FDR correction method was used when multiple comparisons were performed; p < 0.05 was considered statistically significant.

## Results

Data from 29 SSc patients (27 females (93%), 2 males) and 20 HCs (14 females (70%), 6 males) are shown in Table 1. Thirteen SSc patients were diagnosed with SIBO (44.8%). Heartburn (reflux), distension and bloating (D/B), fecal incontinence (soiling), diarrhea, constipation, social functioning (SF), and emotional well-being (EW) were reported from the GIT 2.0 questionnaire.

**Table 1.** Characteristics of GI symptoms in patients with SSc characterized by the UCLA SCTC GIT 2.0.

	SIBO+ (n = 13)	SIBO− (n = 16)	SSc participants (n = 29)	Healthy controls (n = 20)
Female, n (%)	12 (92)	15 (94)	27 (93)	14 (70)
Mean (SD) age, years	56.9 (11.6)	54.3 (11.2)	55.5 (11.2)	33.1 (13.5)
Mean (SD) BMI, kg/m^2^	26.2 (5.9)	24.5 (2.1)	25.2 (4.2)	25.0 (3.5)
SSc subtype, n (%)^a^				
dcSSc	2	5	7	
lcSSc	10	10	20	
ssSSc	0	1	1	
ACA+, n (%)	11 (85)	9 (56)	20 (69)	N/A
Mean (SD) SSc duration, years	11.2 (7.4)	14.7 (14.2)	14.4 (11.6)	N/A
GERD, n (%)	5 (38)	3 (19)	8 (28)	N/A
On immunosuppression, n (%)	4 (31)	7 (44)	11 (38)	N/A
Mean (SD) GIT 2.0 scores				N/A
Reflux	0.59 (0.56)	1.21 (0.64)	0.90 (0.67)	
D/B	1.27 (0.77)	1.52 (0.66)	1.36 (0.73)	
Soilage	0.62 (0.87)	0.4 (0.51)	0.48 (0.69)	
Diarrhea	0.62 (0.46)	0.43 (0.42)	0.50 (0.44)	
Constipation	0.65 (0.45)	0.85 (0.50)	0.73 (0.49)	
SF	0.56 (0.52)	0.72 (0.61)	0.62 (0.57)	
EW	0.60 (0.71)	0.56 (0.72)	0.56 (0.70)	
Total	0.71 (0.48)	0.81 (0.35)	0.74 (0.42)	

Factors such as reflux (heartburn), distension/bloating (D/B), fecal soilage (incontinence), diarrhea, constipation, social functioning (SF), and emotional well-being (EW) were measured as part of the UCLA GIT 2.0 questionnaire.

UCLA: University of California Los Angeles; SCTC: Scleroderma Clinical Trial Consortium; GIT: gastrointestinal tract; SIBO: small intestinal bacterial overgrowth; SSc: systemic sclerosis; SD: standard deviation; BMI: body mass index; dcSSc: diffuse cutaneous scleroderma; lcSSc: limited cutaneous scleroderma; ssSSc: systemic sclerosis sine scleroderma; ACA: anticentromere antibody Anticentromere Antibody; N/A: not applicable; GERD: gastroesophageal reflux disease.

a Data included for 28 study participants.

### The microbiome of SSc patients differs from HCs

**Figure 1.**
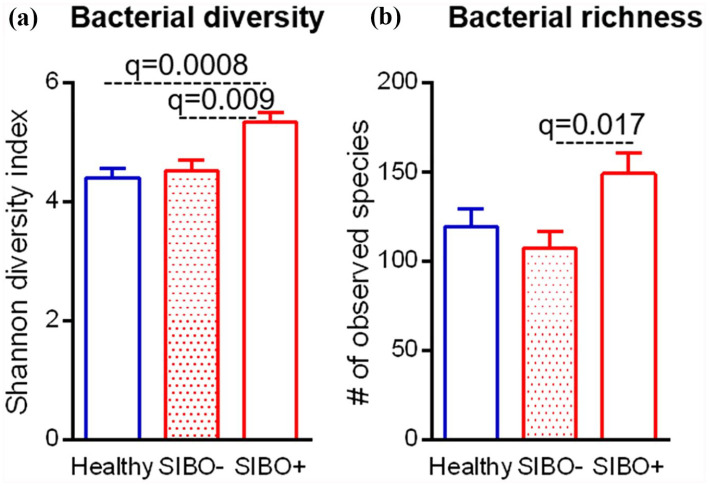
Alpha diversity measures of HCs and patients with SSc. (a) Shannon diversity index and (b) number of observed species of HCs and SSc patients with and without small intestinal bacterial overgrowth. The data were analyzed with Kruskal–Wallis test, followed by Dunn’s multiple comparison test.

**Figure 2.**
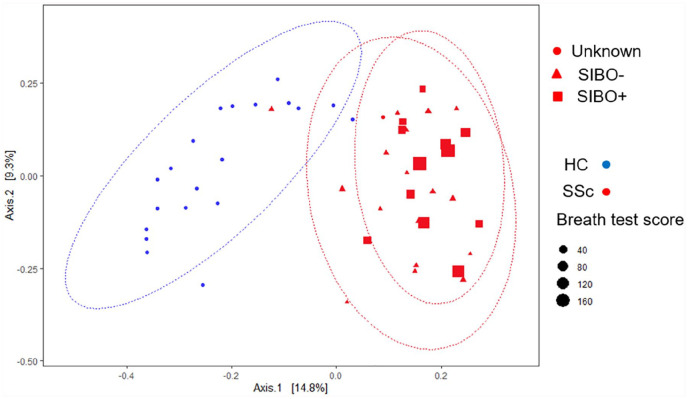
Beta diversity in healthy controls (HCs) and patients with scleroderma (SSc). Principal coordinate analysis of Bray Curtis dissimilarity matrix. SSc patients presented with a significantly different gut microbiota (Multiple Response Permutation Procedure (MRPP) p = 0.001). The size of the dots is proportional to the results obtained from the SIBO hydrogen breath test. The ellipses constructed around samples delimit the statistical place of each cluster, assuming a multivariate *t*-distribution.

The fecal microbiota composition of patients and HCs was compared (Figure 1). Alpha diversity, measured with the Shannon Diversity Index (SDI), measures both species numbers and their abundance equality, with larger values indicating many species with well-balanced abundances. In our cohort, alpha diversity differed between patients and HCs (q = 0.017), but no differences in microbiome richness (i.e. absolute number of observed species) were observed. Beta diversity, assessed by the Bray Curtis dissimilarity matrix (Figure 2), demonstrates differences between different microbial communities from different environments. In our study, beta diversity differed between HCs and SSc patients (p = 0.001).

### Microbiota characterization of SSc patients versus HCs

**Figure 3.**
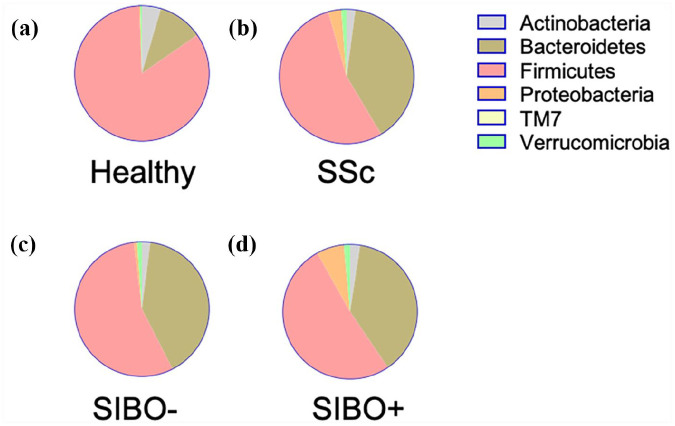
Taxonomic compositions of fecal samples at phylum level for (a) HCs. (b) SSc patients, further divided into those without (c) SIBO and with (d) SIBO.

**Figure 4.**
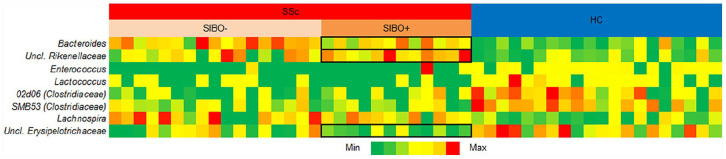
Fecal taxonomic composition at genus level of bacterial genera significantly different between SSc patients with SIBO and SSc patients without SIBO and HCs (HC). The heatmap depicts significant statistical comparisons between SSc patients and HCs while also showing whether a patient presented with SIBO or not. Each column corresponds to one patient. In general, SIBO+ SSc patients had significant changes in alpha diversity when compared to healthy controls, with genera outlined in black squares indicating which taxa were significantly different in relative abundance. In addition, SIBO+ SSc patients exhibited more overall diversity and richness than SIBO− SSc patients. The data were analyzed with the two-tailed Mann–Whitney U-test, followed by Benjamini–Hochberg false discovery rate (FDR) multiple comparison correction (α ⩽ 0.05).

At the phylum level (Figure 3), SSc patients exhibited a higher relative abundance of Proteobacteria (q = 0.002) and Bacteroidetes (q = 0.0007) and lower abundance of Firmicutes (q = 0.001). Indeed, the Firmicutes/Bacteroidetes ratio was substantially lower in patients with SSc (*p* < 0.0001) (25 compared to 3). At the genera level (Figure 4), SSc patients as a whole exhibited lower relative abundance of *Enterococcus* spp. (q = 0.0002), *Lactococcus* spp. (q = 0.0002), *02d06* spp. (q = 0.0003), and *SMB53* spp. (q = 0.0003). Higher levels of relative abundances of *Bacteroides* (q = 0.00005) and *Lachnospira* (q = 0.0006) were also observed. When comparing the influence of ACA status on the microbiota of SSc, no differences were found.

### Effect of SIBO on the microbiota composition of SSc patients and HCs

When comparing alpha diversity between HC and specifically SSc patients with SIBO, only significant differences in bacterial diversity, but not richness, were found (q = 0.008). There was a significantly larger relative abundance of *Bacteroides* spp. (q = 0.0001) and *Uncl. Rickenellaceae* spp. (q = 0.0001) in SIBO+ SSc patients compared to HC (Figure 4). In addition, significantly smaller relative abundance was found in *Uncl. Erysipelotrichacaea* spp (q = 0.0003) in SIBO+ SSc patients compared to HC. No significant differences were found between HC and SIBO- SSc patients. When observing SSc patients with SIBO versus without SIBO, differences were found in both diversity (q = 0.0086) and richness (q = 0.0032; Figure 1).

### Correlation of ACA, SCL-70 status, and specific bacteria with GI symptoms in SSc patients

**Figure 5.**
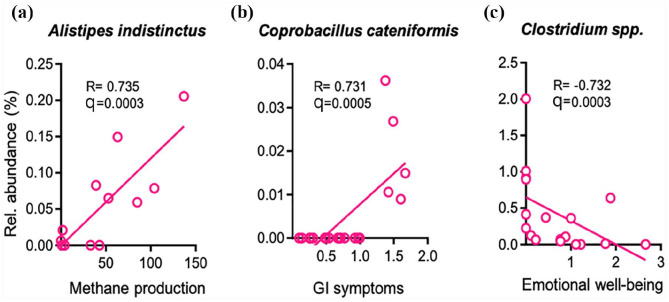
Relative abundance (%) of bacterial species in anticentromere antibody (ACA)-positive SSc patients and their (a) methane production, (b) GI symptoms, and (c) emotional well-being.

No significant correlations between antibody status and microbiota composition were found among the entire cohort, including ACA+, ACA−, SCL-70+ patients. In ACA− or SCL-70+ patients, no significant associations were found with breath testing or specific bacteria. However, in ACA+ patients, four correlations were identified. First, a higher relative abundance of *Alistipes indistincus* was positively associated (R = 0.735, q = 0.0003) with methane (CH_4_) levels on breath gas testing (Figure 5A). A higher relative abundance of *Coprobacillus cateniformis* was associated (R = 0.731, q = 0.0005) with increased overall GI symptoms (Figure 5B). A lower relative abundance of *Clostridium* spp. was associated with worse emotional well-being (Figure 5C). A higher relative abundance of *Slakia* spp was associated (R = 0.708, q = 0.0003) with higher rates of fecal soiling.

## Discussion

In this work, we report a difference in the fecal microbiota composition of Canadian patients with SSc in comparison to HCs, including differences in bacterial diversity and richness. We are the first to report differences in the microbiomes of patients with SSc with and without confirmed SIBO, highlighting taxa altered and associated GI symptoms in these patients.

While the cause of GI symptoms in patients with SSc is unclear, the unique microbiome of these patients is suggested to play a role.^28^ The challenge with this has been determining whether pathological changes in the GI tract and subsequent gut dysfunction lead to an altered microbiome, or if the microbiome itself triggers the development of future fibrosis.^15,18^ Furthermore, while SIBO is highly prevalent (39%) in patients with SSc,^29^ to date, no study has investigated the microbiome in SSc patients with and without SIBO.

In our study, microbial diversity was greater in patients with SSc than in HCs, but microbial richness, a measure of the absolute number of species in a sample, was similar between SSc patients and HCs. This suggests that while the number of species vary, healthy subjects have microbiomes more homogeneous in nature. Lower microbial diversity was previously reported in SSc patients by four other studies, including one colonic lavage study (UCLA)^30^ and three independent SSc stool sample analyses (UCLA, Oslo, Milan).^13,31^ It is possible that our study differs due to inclusion of patients affected by SIBO, which present with overgrowth of bacteria that could easily shed, affecting the composition of the lower GI tract and feces. This is further supported by the fact that there were no differences in richness or diversity between SIBO− and HCs. The beta diversity between HC and SSc patients differed, in agreement with some previous SSc cohorts (UCLA, Oslo),^11,13^ but contrary to others (Milan).^31^ While age discrepancy between SSc and HCs was thought to play a role in the observed differences, when age-corrected, the results did not change, excluding this as a contributing factor. These findings provide further support that patients with SSc have distinctly different microbiomes from their healthy counterparts.

Similar to previous work in SSc and other autoimmune disorders,^13,32,33^ we showed a reduction in the relative abundance of Firmicutes and an increase in Bacteroidetes for SSc patients. This resulted in a significant decrease in the ratio of Firmicutes:Bacteroidetes in these patients. This ratio is relevant as it has been postulated to play an important effect on human health,^33^ and perhaps the development of autoimmune pathologies. Patients with systemic lupus erythematosus have demonstrated similar decreases to the Firmicutes:Bacteroidetes,^34^ highlighting the need for a comprehensive understanding of how this ratio applies to autoimmune conditions, both selectively and as a whole.

Moreover, SSc patients exhibited a lower relative abundance of certain lactic acid bacteria (LAB), such as *Enterococcus* and *Lactococcus*, a result not previously found in an SSc cohort. While increased abundance of *Enterococci* is known to correlate with symptoms of SIBO,^35^ the decreased abundance of *Enterococcus* and *Lactococcus* seen in our cohort might impact GI symptoms. Previous works have shown that administration of lactobacillus reduces autoimmune GI symptoms,^36^ allergies,^37^ and improves both the innate and adaptive immune response. Furthermore, as both *Enterococcus* and *Lactococcus* are LAB, their combined decrease in patients with SSc likely reflects a shift in the fecal microenvironment. Such a shift is perhaps due to the large proportion (41%) of SSc patients with SIBO and the potentially downstream effect this overgrowth has on the fecal microbiota.

Our results demonstrated higher relative abundance of commensal *Bacteroides* spp. in SSc patients in comparison to HCs. These are commensals that have been shown to negatively affect GI symptoms in SSc over time when in low abundance.^38^ However, *Bacteroides* spp. are known to be responsible for many infections, with the ability to evade the host immune system contributing to its virulence.^39^ Thus, a better understanding of this genus and its clinical relevance are important when considering future therapies for GI symptoms in SSc patients. Despite our results contrasting literature, two previous cohorts (Oslo, Los Angeles) exhibited significant differences in *Bacteroides* levels, which likely reflects the impact of genetic, environmental, and dietary influences on its abundance. These factors have previously been shown to affect levels of *Bacteroides*^40^ and the microbiome as a whole.^16^

When taking into account the effect of SIBO on the microbiome, SIBO+ patients demonstrated increased bacterial diversity and richness when compared to SIBO− SSc patients, and only increased diversity when compared to HC. This finding in SSc patients was expected, given the diagnostic criteria (>105 colony-forming units or the presence of atypical bacteria) of SIBO. However, the difference observed when compared to HC may signify a role for microbiota depletion in SSc patients. At the phylum level, SIBO+ patients exhibited increases in the relative abundance of *Proteobacteria* when compared to SIBO− SSc patients, which was also observed in another SSc cohort where the SIBO status of participants was not determined.^30^ *Proteobacteria* have been associated with pro-inflammatory states,^17^ such as IBD.^33^ The increased abundance of *Proteobacteria* in SIBO+ SSc patients provides evidence that microbiome changes in the small bowel can affect the downstream (fecal) microbiome, with increases in potentially pathogenic bacteria. At the genera level, SIBO+ SSc patients exhibited significant differences in the relative abundance of certain genera with a higher relative abundance of *Bacteroides* and *Uncl. Rikenellaceae* and lower relative abundance of *Uncl. Erysipelotrichaceae* compared to HCs. This may influence the development of SSc, with a higher *Rikenellaceae* abundance found in ankylosing spondylitis patients^41^ and lower levels of *Erysipelotrichaceae* found in patients with new and recurrent Crohn’s disease.^42^ While no definitive conclusions can be made, these findings provide further evidence that dysbiosis itself may play a role in the development of SSc.

There were no significant differences found at the genus level regardless of SIBO status, a finding which may reflect the relatively small number of SIBO+ patients. Furthermore, ACA status did not appear to affect the microbiota of SSc patients versus HC, nor did it influence microbiota results in SIBO+ versus SIBO− patients; however, a larger cohort is required to make definite conclusions. Previous studies have shown no differences observed in the GI manifestations of SSc between ACA-positive and ACA-negative SSc patients.^43,44^ Furthermore, in previous works, no associations with ACA status were found to impact the microbiomes of patients,^28^ nor did it vary with SIBO status.^14^ Bearing in mind that while ACA status is a useful tool in the diagnosis of SSc, and most patients included in our study were ACA-positive, ACA status still does not appear to predict the degree of GI involvement/symptoms or SIBO status. In addition, while SIBO− SSc patients had higher GIT scores, symptoms were mostly similar between SIBO+ and SIBO− patients. The total score was slightly higher in SIBO− patients and was driven mainly by reflux and constipation. As these two symptoms are highly associated, this likely reflects the overall role of slower GI motility. Conversely, SIBO+ patients had higher scores for diarrhea and soilage, which are likely consequences of faster GI transit time.

This study has several limitations. First, the nature of this cross-sectional study did not allow us to analyze the microbiome over time or relative to disease changes. Second, our sample size was not age-matched to controls, ethnicity, diet, and lifestyle habits. However, significant differences were seen between those with and without SSc even after accounting for age. Third, we did not collect data on interstitial lung disease (ILD) in our patients, which may influence certain microbiome profiles.^28^ Finally, our microbial samples were obtained from fecal samples, and thus our results may differ from what would be seen with mucosal sampling. Despite these limitations, our study is the first to report differences in the microbiomes of Canadian patients with SSc and in SIBO+ versus SIBO− patients with SSc.

In conclusion, this study identified unique microbiota profiles in patients with SSc in comparison with HCs, as well as differences in microbial composition in SIBO-positive versus SIBO-negative SSc patients. Furthermore, we have found novel associations between certain bacterial taxa and emotional well-being in SSc. Taken together, these findings improve our understanding of the fecal microbiome’s role in SSc, providing another puzzle piece in determining the complex etiology of SSc and co-morbid GI symptoms. While a larger sample size is needed to validate these results, they provide potential bacterial targets for therapies aimed at improving the GI symptoms and quality of life in patients with SSc.

The authors of this article do not report any financial support or other benefits from commercial sources for the work reported in the article. The authors also report no other financial interests, which could create a potential conflict of interest or the appearance of a conflict of interest with regard to the work.

## Supplemental Material

sj-pdf-1-jso-10.1177_23971983211032808 – Supplemental material for Fecal microbiome differs between patients with systemic sclerosis with and without small intestinal bacterial overgrowth Click here for additional data file. Supplemental material, sj-pdf-1-jso-10.1177_23971983211032808 for Fecal microbiome differs between patients with systemic sclerosis with and without small intestinal bacterial overgrowth by Daniel Levin, Giada De Palma, Hannah Zou, Ava HD Bazzaz, Elena Verdu, Barbara Baker, Maria Ines Pinto-Sanchez, Nader Khalidi, Maggie J Larché, Karen A. Beattie and Premysl Bercik in Journal of Scleroderma and Related Disorders
