# Supplementary material for: Fecal microbiome differs between patients with systemic sclerosis with and without small intestinal bacterial overgrowth
Source: J Scleroderma Relat Disord. 2021 Jul 24;6(3):290–8. doi: 10.1177/23971983211032808 (PMC8922657; doi:10.1177/23971983211032808)
Supplement: sj-pdf-1-jso-10.1177_23971983211032808 – Supplemental material for Fecal microbiome differs between patients with systemic sclerosis with and without small intestinal bacterial overgrowth [file sj-pdf-1-jso-10.1177_23971983211032808.pdf]

St. Joseph's Healthcare Hamilton McMaster University

**Instructions for Preparing for the Breath Test**

- No eating or drinking (water is okay) for 12 hours prior to the test. (Diabetic patients should consult with their provider prior to fasting to ensure this test is safe for them.)
- 2 WEEKS BEFORE YOUR TEST
  - Finish taking any antibiotics or antifungals. Do not undergo colonoscopy or barium enema during this time (regular home enemas like Fleet are ok).
- 7 DAYS BEFORE YOUR TEST
  - Unless otherwise directed by your provider, please avoid taking any Proton Pump Inhibitors (PPIs). If absolutely necessary, you may take your PPI **except** the day of the test.
- 4 DAYS BEFORE YOUR TEST
  - Avoid all laxatives including Vitamin C and Magnesium.
- 1-2 DAYS BEFORE YOUR TEST
  - Do not eat foods containing high fibre or lactose. These foods can ferment in your gut, and can potentially affect the results. If you have constipation, please refrain for the full two days.
  - The following foods <u>are</u> acceptable:
    - Baked or boiled chicken, fish or turkey
    - Plain, steamed white rice (If you are on a grain free diet, please do not consume rice)
    - Eggs
    - Clear meat broth (no bouillon, bone/cartilage or vegetable broth)
    - Fats/oils (e.g., coconut/olive/vegetable oil, butter, lard)
    - Salt and pepper
    - Weak black coffee/tea (plain, no sweeteners or cream)
      - **If you are unsure if something will interfere with the test, DO NOT CONSUME the product and/or consult with your provider**
- 12 HOURS BEFORE YOUR TEST
  - Begin fasting. Avoid all food, drinks (except water) and non-essential medication. Please do not take a laxative from now until the test is complete.
- THE DAY OF YOUR TEST
  - **You may brush your teeth as normal. No smoking or vigorous exercise for at least one hour prior to collection. You may wish to bring something to occupy your time while you are having the test (reading material, laptop, iPad, etc.).**

Supplementary Materials 1: Preparation instructions given to patients before breath testing.
